# Medication Use in Early-HD Participants in Track-HD: an Investigation of its Effects on Clinical Performance

**DOI:** 10.1371/currents.hd.8060298fac1801b01ccea6acc00f97cb

**Published:** 2016-01-11

**Authors:** Ruth Keogh, Chris Frost, Gail Owen, Rhian M. Daniel, Doug R. Langbehn, Blair Leavitt, Alexandra Durr, Raymund A.C. Roos, G. Bernhard Landwehrmeyer, Ralf Reilmann, Beth Borowsky, Julie Stout, David Craufurd, Sarah J. Tabrizi

**Affiliations:** Medical Statistics, London School of Hygiene and Tropical Medicine, London, UK; Medical Statistics, London School of Hygiene and Tropical Medicine, London, UK; UCL Institute of Neurology, University College London, Queen Square, London, UK; Medical Statistics, London School of Hygiene and Tropical Medicine, London, UK; Psychiatry, University of Iowa, Iowa City, Iowa, USA; Medical Genetics, University of British Columbia, Vancouver, British Columbia, Canada; Genetics, APHP- Groupe Hospitalier Pitié-Salpêtrière, Paris, France; INSERM U1127, CNRS UMR7225, UPMC University Paris VI UMR_S1127, Institut du Cerveau et de la Moelle, Paris, France; Neurology, Leiden University Medical Centre, Leiden, Netherlands; Neurology, Ulm University, Ulm, Germany; George-Huntington-Institute, Neurology, University of Münster, Münster, Germany; CHDI Management/CHDI Foundation, Princeton, NJ, USA; School of Psychological Sciences, Monash University, VIC, Australia; Institute of Human Development, University of Manchester; Manchester Academic Health Science Centre; Manchester Centre for Genomic Medicine, Central Manchester University Hospitals NHS Foundation Trust, Manchester, UK; Neurodegenerative Disease, UCL Institute of Neurology, London, UK

## Abstract

Insufficient evidence exists to guide the long-term pharmacological management of Huntington’s disease (HD) although most current interventions rely on symptomatic management. The effect of many frontline treatments on potential endpoints for HD clinical trials remains unknown. Our objective was to investigate how therapies widely used to manage HD affect the symptom for which they are prescribed and other endpoints using data from TRACK-HD. We used longitudinal models to estimate effects of medication use on performance on tests of motor, cognitive and neuropsychiatric function using data from 123 TRACK-HD stage 1/2 participants across four study visits. Adjustment for confounding by prior medication use, prior clinical performance, concomitant use of other medications, and baseline variables (sex, disease group, age, CAG, study site, education) enabled a closer-to-causal interpretation of the associations. Adjusting for baseline variables only, medication use was typically associated with worse clinical performance, reflecting greater medication use in more advanced patients. After additional adjustment for longitudinal confounders such “inverse” associations were generally eliminated and in the expected directions: participants taking neuroleptics tended to have better motor performance, improved affect and poorer cognitive performance, and those taking SSRI/SNRIs had less apathy, less affect and better total behaviour scores. However, we uncovered few statistically significant associations. Limitations include sample size and unmeasured confounding. In conclusion, adjustment for confounding by prior measurements largely eliminated associations between medication use and poorer clinical performance from simple analyses. However, there was little convincing evidence of causal effects of medication on clinical performance and larger cohorts or trials are needed.

## 1. Introduction

Huntington’s disease (HD) is a devastating neurological disorder caused by a dominantly inherited CAG repeat expansion in the huntingtin gene. HD has diverse and progressive symptoms and is characterised by deteriorating motor and cognitive functions, as well as behavioural and neuropsychiatric disturbances. White-matter atrophy is detectable in the earliest premanifest stages of the disease and caudate, putamen, and grey-matter volumes have strong predictive value for future clinical diagnosis of HD in those carrying the gene mutation^1^^,^ 2. However, despite the prognostic value of these structural measures, it is clearly the associated cognitive decline and behavioural changes that present the most challenging features of the disease for HD patients and their families.

The identification of targets for potential disease-modifying therapies is the focus of considerable research effort, but most current interventions rely on symptomatic management of HD (see Videnovic 2013^3^ for a summary). Insufficient clinical evidence exists to guide the long-term pharmacological management of HD. Currently the only FDA-approved treatment for HD is tetrabenazine, which was endorsed for the management of chorea in 2008. Otherwise, treatment of motor dysfunction is largely confined to neuroleptics (e.g. risperidone, olanzapine and quetiapine), some of which may also help to control severe neuropsychiatric symptoms such as delusions, hallucinations and violent outbursts. Other behavioural and neuropsychiatric indications are more commonly managed with serotonin reuptake inhibitors (SSRIs, e.g. fluoxetine, citalopram and paroxetine) and serotonin-norepinephrine reuptake inhibitors (SNRIs, e.g., duloxetine and venlafaxine), and other drugs which increase central noradrenergic and serotonergic neurotransmission (e.g. mirtazapine).

Paradoxically, many of the drugs used in the symptomatic management of HD have side effects that are difficult to distinguish from the progressive symptoms of the disease. Tetrabenazine, for example, carries a risk of potentially serious adverse reactions and may increase the risk of depression^4^, whereas neuroleptics and SSRI/SNRIs may be associated with fatigue, restlessness, anxiety and hyperexcitability. Use of such pharmacological agents may therefore be prohibited before or during future clinical trials, and at the very least their potential effect on a study’s endpoints must be understood in order to establish independent effects of concomitant and study therapies.

Evidence suggests that some pharmacological treatments for symptoms in HD can have unintended effects on potential study endpoints. For example, nearly all clinical trials in HD to date have used the Unified Huntington's Disease Rating Scale total motor score (UHDRS-TMS) and total functional capacity (UHDRS-TFC) measures^5^ as either primary or secondary outcomes. Yet components of the UHDRS, and in particular chorea, are known to improve with tetrabenazine^4^ and neuroleptics, such as clozapine^6^ and tiapride^7^. Candidate therapeutics for cognitive dysfunction are in development^8^, but even less is known about how frontline symptomatic treatments affect cognitive manifestations of HD. Neuroleptic use has been linked with poorer recognition of facial emotions, whereas SSRI use was associated with improved recognition^9^. In the few trials in HD that have included endpoints such as attention, memory and executive function^10^^,^ 11 ^,^ 12, the extent to which concomitant medication was controlled varied, and only one excluded participants who had used cholinergic/anticholinergic/antidopaminergic drugs within 4 weeks of enrolment. None of the trials demonstrated any significant improvement in cognition. Furthermore, there are few studies of the association between neuroleptics and neuropsychiatric symptoms in HD^3^, but evidence from other fields suggests there may be modest improvements in neuropsychiatric symptoms, e.g. in AD^13^^,^ 14. However, these are often accompanied by increases in adverse events, and concerns about increased mortality associated with their long term use have emerged^15^.

For this study, we evaluated associations between use of neuroleptics and commonly used antidepressants, common therapeutic strategies for HD, and measures of motor function, cognition, neuropsychiatry and emotion recognition in individuals in the early stages of HD using 36 months of data from TRACK-HD^1^^,^ 2 ^,^ 16 ^,^ 17. TRACK-HD is a longitudinal, natural history study examining disease progression in individuals with premanifest and early-stage HD and each annual assessment included a battery of potential clinical and biological outcome measures, as well as a detailed history and record of ongoing medication use. Not surprisingly, medication use was highest among the manifest HD participants and the most commonly prescribed drugs in this group were neuroleptics, SSRIs and SNRIs. We focus on use of these most commonly used medications in this paper.

Randomized controlled trials are the gold standard for establishing potentially causal relationships between use of treatments and endpoint measures. However, most allow the use of some medications, particularly antidepressants, so it is important to understand the impact of such concomitant medications on trial endpoints. In the absence of randomized controlled trials, observational data may be used to study the effects of treatments on endpoints of interest. In order to attempt to establish potentially causal effects of treatments on outcomes from observational data we must account for the lack of randomization to treatments. This is done by careful control for variables which are associated with both medication use and the endpoint of interest. There is a growing literature on the use of observational data to estimate the causal effects of treatments^18^. For example observational data has been used to study the causal effects of Zidovudine on CD4 count in individuals with HIV^19^ and that of postmenopausal hormone therapy on coronary heart disease in women^20^.

The aim of our investigation was to use observational data from the TRACK-HD study to estimate, as far as possible, the causal effect of medication on motor function, cognition, neuropsychiatry and emotion recognition (a subset of the TRACK-HD assessments). Since functionality may affect whether a medication is prescribed by the participant’s doctor, prior measures of clinical performance were considered as potential confounders, in the sense that they may serve as indirect indicators of functional decline. Prior medication use was also considered in our analysis as this in turn may affect function and performance of the TRACK-HD assessments. Although these issues presented a challenge for the statistical analysis, the longitudinal nature of the TRACK-HD study data made it possible to perform an analysis that adjusts for potential confounding variables measured in the past (`prior measures’).

## 2. Methods

**2.1 Participants**

The inclusion criteria for TRACK-HD have been described previously^1^. Participants attended annual visits (2008-2011) at four study sites in London (UK), Paris (France), Leiden (Netherlands), and Vancouver (Canada), and 123 early-stage HD individuals were included. Our analysis excluded the TRACK-HD premanifest cohort as medication use in this group was relatively low and the early HD patients represent the most likely participants in imminent clinical trials. Using the UHDRS^5^, early HD participants were designated at baseline as either stage 1 (TFC 11-13) or stage 2 (TFC 7-10)^21^. The study was approved by local ethics committees and written informed consent was obtained from each participant before enrolment.

**2.2 Medication use**

In TRACK-HD, drug name, indication, dose, regimen, frequency and route were recorded together with start and stop dates for each medication. The most commonly prescribed medications were neuroleptics, and serotonergic antidepressants (SSRIs, SNRIs and related compounds or both, referred to henceforth as SSRI/SNRI). Only a very small number of individuals were recorded to be using tetrabenazine in this study and so we excluded this from our analyses. Our analysis therefore focuses on neuroleptics and SSRI/SNRIs and Supplementary Table 1 catalogues the specific agents in use for each class of medication. Start and stop dates were used to identify whether a given medication was being taken at each participant study visit.

**2.3 Clinical performance assessment**

TRACK-HD included a range of clinical, cognitive, quantitative motor (Q-Motor), and neuropsychiatric assessments; significant cross-sectional and longitudinal differences in performance between the early-HD group and controls have been reported previously^1^^,^ 2 ^,^ 16 ^,^ 17 and are not discussed here. The individual tests below were selected from the TRACK-HD assessments on the basis that they have been reported on previously^1^^,^ 2 ^,^ 16 ^,^ 17, were measured at all participant visits and have been found to change significantly over time in early HD individuals relative to controls or to be associated with progression over time in TFC in early HD individuals. The UHDRS measures were also selected because they are used as primary outcomes in randomized controlled trials in HD.

Motor function was assessed by total motor, chorea, oculomotor, and bradykinesia scores from the UHDRS^5^. Motor performance was also assessed quantitatively using a Q-Motor battery, which included chorea position and chorea orientation indices (Choreomotography, assessing involuntary choreatic movements), and variability in grip force (Manumotography), isometric tongue protrusion force (Glossomotography), and speeded tapping (Digitomotography).

Cognitive performance was measured using the Symbol Digit Modalities Test (SDMT; number correct), Stroop Word Reading condition (number correct), Direct and Indirect Circle Tracing (annulus length), and a visual working memory task known as Spot the Change (set size 5; number correct). The cognitive battery also included a test of recognition of facial expressions of emotions. We used the scores for the number of correctly identified following emotions: anger, disgust, fear, happiness, neutral, sadness, surprise.

Neuropsychiatric symptoms were identified during a structured interview using a shortened version of the Problem Behaviours Assessment (PBA-s)^22^. Depression, suicidal ideation, anxiety, irritability, aggression, apathy and perseveration were scored on a five‐point (0‐4) scale for severity and a five‐point scale for frequency (over the course of the previous four weeks). The severity and frequency scores were then multiplied to yield an overall score for each symptom, which were then added to produce composite scores for affect (sum of scores for depression, suicidal ideation and anxiety) irritability (sum of scores for irritability, anger and aggression, and perseveration), apathy and a total behavioural score (sum of scores for depression, suicidal ideation, anxiety, irritability, anger and aggression, apathy and perseveration). In the analyses outlined below, we also made use of estimates of the worst levels of severity of affect, irritability and apathy in the preceding year; these are referred to as the ‘secondary neuropsychiatry scores’. In addition we included the Hospital Anxiety/Depression Scale (HADS) total score.

**2.4 Statistical methods**

We let \begin{equation*}X_t\end{equation*} denote medication use (either neuroleptics or SSRI/SNRI) at study visit \begin{equation*}t\end{equation*} \begin{equation*}(t=1,2,3,4)\end{equation*} ; this is a binary variable taking value 1 if an individual is taking the medication at visit \begin{equation*}t\end{equation*} and value 0 otherwise. \begin{equation*}Y_t\end{equation*} denotes the task performance measure of interest as the outcome at study visit \begin{equation*}t\end{equation*} . \begin{equation*}L_t \end{equation*} denotes the collection (i.e. a vector) of all other task performance measures at study visit \begin{equation*}t\end{equation*} that we need to take account of in a study of the causal effect of \begin{equation*}X_t\end{equation*} on \begin{equation*}Y_t\end{equation*} . Baseline variables are denoted by \begin{equation*}Z\end{equation*} , which contains sex, disease subgroup (stage 1 or stage 2), age and CAG and their interaction, study site, education level (six categories). A bar over a variable at time \begin{equation*}t\end{equation*} denotes the history of measures of that type up to time , e.g. \begin{equation*}\bar X_t =\{X_1,X_2, \ldots  ,X_t\}\end{equation*} . Under the models described below, the task performance measure is the outcome or response variable and medication use is the main exposure of interest.

We started by considering Model 1 in which only baseline variables are included as potential confounders of the association between medication use at time t and clinical performance at time \begin{equation*}t\end{equation*}

**Model (1):**

\begin{equation*}E(Y_t|X_t,Z)=\theta+\phi X_t+\psi^T Z\end{equation*}

Under this model, the expected outcome at time \begin{equation*}t\end{equation*} (\begin{equation*}Y_t\end{equation*}) is modelled as a function of concurrent medication use (\begin{equation*}X_t\end{equation*}) and baseline variables (\begin{equation*}Z\end{equation*} ). Parameter φ denotes the association of the exposure with the outcome given baseline variables; that is, the mean difference in the outcome \begin{equation*}Y_t\end{equation*} for individuals taking medication versus not taking medication at a given time, conditional on baseline variables \begin{equation*}Z\end{equation*} . We emphasise that the baseline variables do not include medication status or clinical performance measures at baseline.

Model 1 does not accommodate confounding by prior medication use and prior clinical performance measures. To attempt to come closer to a causal interpretation for the estimates of associations between medication use and clinical performance measures we need to control for the potential roles of these other variables. Directed acyclic graphs (DAGs) or ‘causal diagrams’ are used to describe the potential longitudinal relationships between medication use and clinical performance. Here we use the causal diagram in Figure 1. This takes into account the possibility that prior clinical performance. i.e. the outcome at the previous visit, may affect whether a patient is prescribed a medication, and vice versa. It also indicates the possible need to adjust for other prior measures of clinical performance – the aim of such adjustment is to help to control for some of the confounding implied by the connections of unmeasured disease status (D) with clinical performance measures and medication use. Use of other medications may also confound associations. Figure 1 also illustrates the need to consider the possibility that there could be unmeasured confounding that we cannot control for in the analysis. Corresponding to Figure 1, the more complex model we fitted, denoted Model 2, is of the form:

**Model (2):**

\begin{equation*}E(Y_t|\bar{Y}_{t-1},\bar{X}_t,\bar{L}_{t-1})=\alpha+\beta Y_{t-1}+\gamma X_t +\delta X_{t-1}+\eta^T L_{t-1}+\tau X_t ^*+\rho^T Z\end{equation*}

where \begin{equation*}X_t ^* \end{equation*} denotes SSRI/SNRI use if \begin{equation*}X_t \end{equation*} denotes neuroleptics use, and vice versa. The variables contained in \begin{equation*}Y_t\end{equation*} , \begin{equation*}X_t\end{equation*} , and \begin{equation*}L_{t-1}\end{equation*} for each type of clinical performance measure and for each medication type are shown in Table 1. Note that we do not necessarily believe that the \begin{equation*}L_{t-1}\end{equation*} causally affect \begin{equation*}Y_t\end{equation*} , but rather that there is an association between these variables via the unmeasured underlying disease status, which is likely to more realistic – this is represented in Figure 1 by showing the unobserved underlying disease states \begin{equation*}D_t\end{equation*} influencing \begin{equation*}L_t\end{equation*} , \begin{equation*}Y_t \end{equation*} and \begin{equation*}X_{t+1}\end{equation*} and therefore inducing confounding by \begin{equation*}L_{t-1}\end{equation*} . This motivates adjusting for \begin{equation*}L_{t-1}\%0A\end{equation*} in Model 2. Inclusion of additional variables, from those considered in this study, in \begin{equation*}L_{t-1}\end{equation*} aside from those listed in Table 1 did not have any material impact on the results. For example, adjustment for cognitive, neuropsychiatric or emotion recognition measures in models for Neuroleptics with a motor function measure as the outcome. We did not consider adjustment for use of medications other than neuroleptics and SSRI/SNRIs due to small numbers of individuals using other medications.

Parameter γ denotes the association of medication use with clinical performance given baseline variables and all other variables in the model. This parameter is represented in Figure 1 by the direct arrows from \begin{equation*}X_t \end{equation*} to \begin{equation*}Y_t\end{equation*} . Note that the model is focused on estimating this particular association and by fitting model (2) we do not directly estimate the parameters associated with the other arrows shown in Figure 1. If there is no unmeasured confounding (as depicted in Figure 1) and the model is correctly specified then the parameter γ is interpreted as the causal effect of medication use on the clinical outcome measure, as measured by a mean difference in the outcome for individuals on medication versus those not on medication. However, causal interpretations should be made with caution because the assumption of no unmeasured confounding may not be met, despite us adjusting for a large number of variables in the analyses.

Models 1 and 2 are fitted using generalised estimating equations (GEEs) using independence working correlation structure^23^^,^ 24. We do not use the data on the association between medication use and clinical performance from visit 1 – this is because we assume that there is unmeasured confounding due to prior measures which are not observed. However, data from visit 1 will still be used to provide adjustment variables for visit 2 associations.

There is some missing data due to drop out from the study and due to some participants not completing a particular assessment at a given study visit. This is summarised in Supplementary Table 2. Models 1 and 2 were fitted first using a complete-case analysis, i.e. dropping any subjects with any incomplete data. Note that Model 2 suffers a greater missing data problem than Model 1 because it includes more explanatory variables. We also used multiple imputation by chained equations^25^^,^ 26 to impute the missing data, using the approach for longitudinal data described by Nevalainen et al (2009)^27^, with 100 imputed data sets. Model 2 was refitted on the imputed data sets and the resulting estimates combined using Rubin’s Rules^25^. All analyses were performed using R. The multiple imputation was performed using the ‘mice’ package28.

**Associations between exposures X (medication use) and outcomes Y (clinical performance measures) at four study visits, including other longitudinal measured variables L and unmeasured ‘underlying disease status’ D, which is assumed to be not completely captured in the measured variables Y and L. Solid-line arrows represent the possible effect of one variable on another. Dotted lines represent possible effects of measured variables on unmeasured disease status, and vice versa. Baseline variables Z, which are not time-varying, are not shown but are assumed to potentially affect all other variables.**
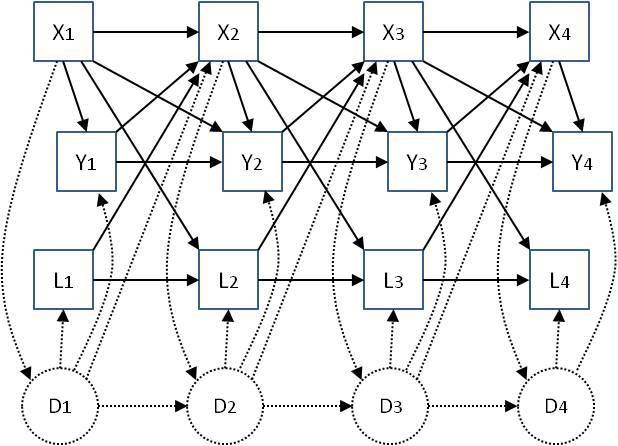

**Figure:**
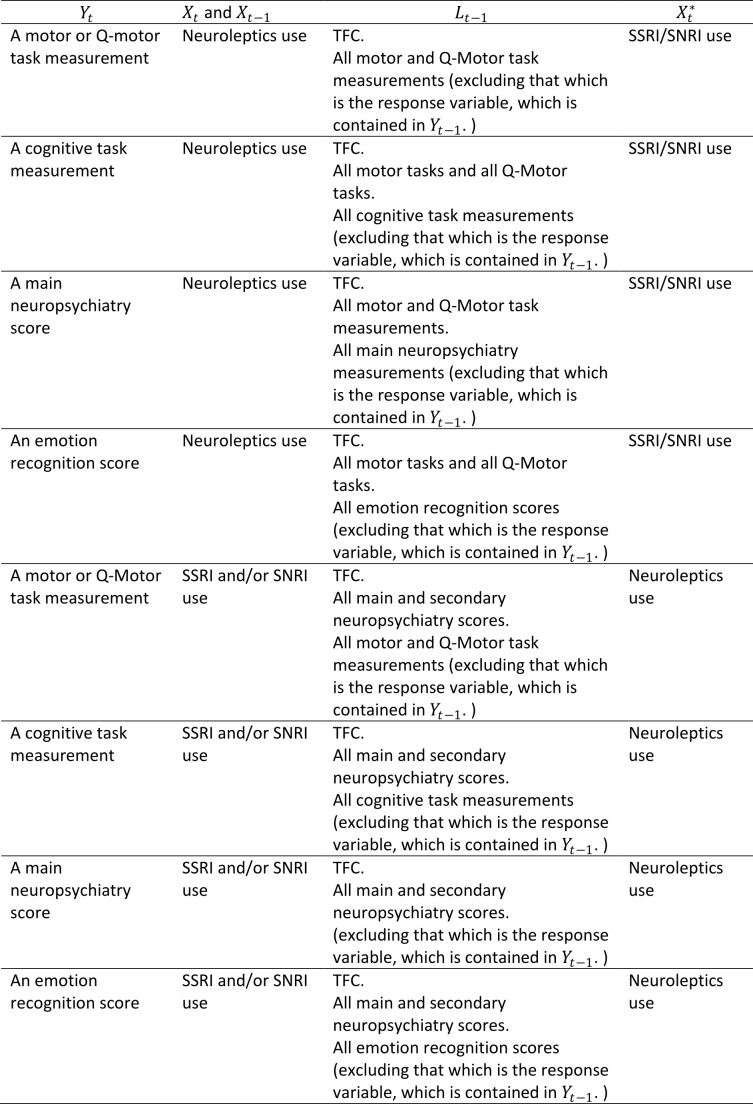
**Table 1:** The variables contained in Model 2 for each type of clinical performance measure and for each medication type.

## 3. Results

**3.1 Summary of medication use**

Table 2 summarises medication use by study visit. Table 3 shows a summary of the baseline characteristics of the early-HD population in TRACK-HD, overall and by medication use (neuroleptics and SSRI/SNRIs).

Use of neuroleptics at baseline was higher in men than women (33.9% versus 17.9%, p-value 0.042). There was a strong association between study site and neuroleptics use (p-value <0.001), with the Paris site having a high proportion of users (56.7%) compared to the London site, which had the lowest proportion (6.7%). A higher level of education was also statistically significantly associated with greater use of neuroleptics (p-value 0.004). No associations were found between neuroleptics use and disease stage, CAG, or age.

A quarter of early-HD participants were users of neuroleptics at baseline (25.2%) and this increased to 42.3% at 36 months (visit 4). Many individuals were continuing users, but there were also 28 new users of neuroleptics over the course of the study. Use of SSRI/SNRIs was higher, increasing from 46.3% at baseline to 55.7% by 36 months, but with fewer new users, at just 18 over the course of the study. At baseline, 13.8% were using both neuroleptics and SSRI/SNRIs, and this increased to 28.9% at 36 months.

The average age of SSRI/SNRI users was significantly higher than in non-users (51 versus 46, p-value 0.007) and those with stage 2 HD were more likely to be taking SSRI/SNRIs than those who were HD stage 1 (54.4% versus 33.7%, p-value <0.001). Use of SSRI/SNRIs did not differ statistically significantly between men and women, by CAG, by study site or by education.

**Figure:**
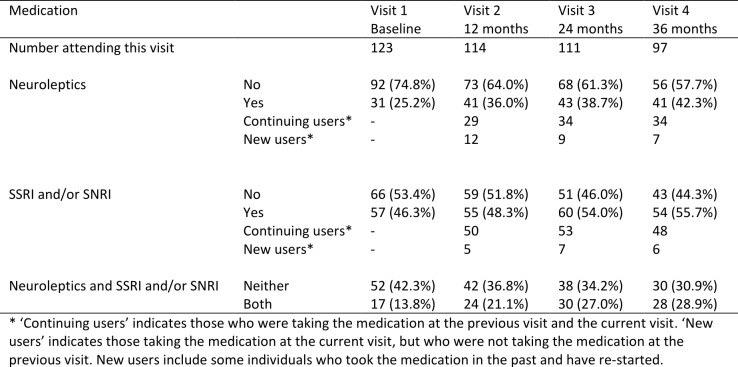
**Table 2:** Summary of medication use among early-HD participants in TRACK-HD. Results are Number or Number (%).

**Figure:**
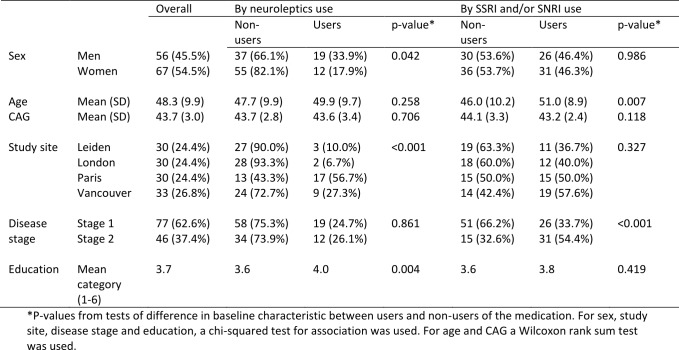
**Table 3:** Summary of baseline characteristics of early-HD participants in TRACK-HD, and associations with medication use at visit 1. Results are Number (%) unless otherwise specified.

**3.2 Effects of medication on clinical performance measurements.**

In the reporting of the results we focus not only on statistical significance but also on the magnitude and direction of the association between medication use and the measures of performance. In particular we consider the impact of adjustment for potential confounding by prior medication use, prior performance measures and use of other medications on the direction of the association.

***Motor***

The estimated associations between medication use and performance on motor tasks are shown in Table 4 (higher scores represent a worse performance). After adjustment for baseline covariates only (Model 1), those using neuroleptics tended to have higher scores on UHDRS-TMS and Q-Motor tasks than those not using neuroleptics. The difference was statistically significant (p-value <0.05) for the UHDRS-TMS, its oculomotor and bradykinesia sub-scores, and for grip force variability, and chorea orientation and position indices. One reason for these results may be that individuals with worse disease status were more likely to take neuroleptics. In Model 2 we made additional adjustment for prior medication use, use of other medications, and prior performance measures, in addition to the baseline variables. In the results from Model 2 we see a reversal of the direction of the association between medication use and some measures of motor performance, in particular for UHDRS-TMS, UHDRS chorea sum score and grip force variability, compared with the results from Model 1. After conditioning on prior medication use, use of other medications, and prior performance measures in addition to baseline covariates, those using neuroleptics had significantly lower UHDRS chorea sum score compared to non-users and total motor score and grip force variability were also slightly improved compared with non-users, though these differences were not statistically significant. The associations between neuroleptic use and other motor and Q-motor performance measures were reduced to approximately zero under Model 2, with the exception of the UHDRS oculomotor sum score, for which the association under Model 2 is the same direction as that under Model 1, but the association is no longer statistically significant. Differences in the results found under Models 1 and 2 are indicative of the existence of arrows between \begin{equation*}Y_{t-1}\end{equation*} and \begin{equation*}X_t\end{equation*} and between \begin{equation*}L_t\end{equation*} and the other variables as shown in Figure 1.

Accounting for missing data using multiple imputation does not have a material impact on the estimate coefficients, although the association between the chorea score and neuroleptic use is weaker and no longer statistically significant.

In Model 1, which adjusts only for baseline covariates, we found positive and statistically significant associations between use of SSRI/SNRIs and several of the measurements of motor performance. All but one of these statistically significant associations disappeared after adjustment for the further variables under Model 2. The speeded tapping mean inter-tap interval remained significantly higher in SSRI/SNRI users, all else being equal, though this association disappeared after accounting for missing data.

**Figure:**
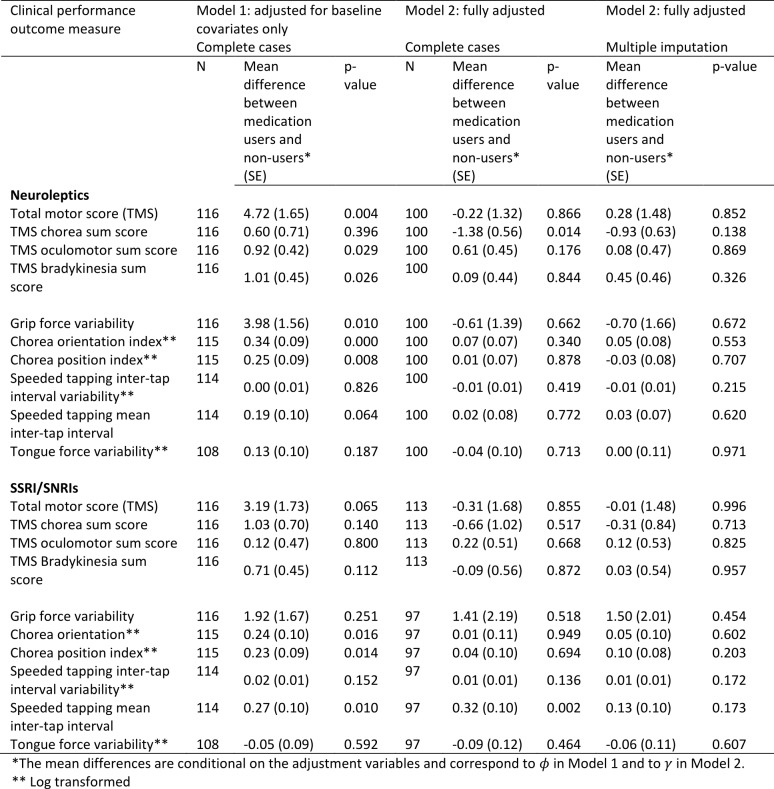
**Table 4:** Associations between medication use and performance on motor tasks and Q-Motor tasks using Model 1 (adjusted for baseline covariates only – sex, disease group, age, CAG, study site, education) and Model 2 (fully adjusted - for baseline covariates, prior medication use, use of other medications, prior performance measures). The models were first fitted on complete cases with no dropout or other missing data (N). Model 2 was then fitted by using multiple imputation to impute missing data.

***Cognition***

The estimated associations between medication use and cognitive scores are shown in Table 5 (higher scores represent a better performance). After adjustment for baseline variables under Model 1, users of neuroleptics had statistically significantly worse performance on all of the cognitive tasks compared with non-users. After adjustment for prior medication use, use of other medications and prior performance measures using Model 2, the associations are in the same direction but are very substantially weakened and rendered non-statistically significant.

Under Model 1, users of SSRI/SNRIs tended to have worse performance on four of the five cognitive tasks, though only one of the associations is statistically significant (Indirect Circle Tracing). After additional adjustment under Model 2, the significant association with indirect circle tracing is no longer evident. For two of the measures, the direction of the association with SSRI/SNRIs is reversed under Model 2. Handling of the missing data using multiple imputation does not materially change the results, though the negative, albeit non-statistically significant, association between SSRI/SNRIs and performance on the Stroop task is markedly attenuated.

**Figure:**
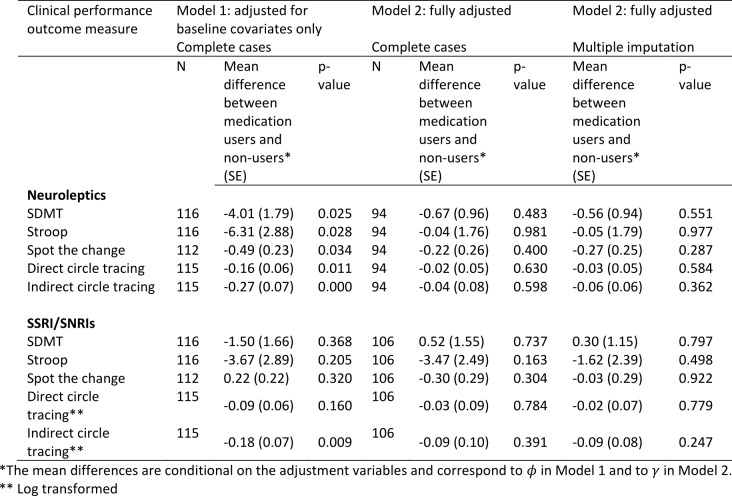
**Table 5:** Associations between medication use and performance on cognitive tasks using Model 1 (adjusted for baseline covariates only – sex, disease group, age, CAG, study site, education) and Model 2 (fully adjusted - for baseline covariates, prior medication use, use of other medications, prior performance measures). The models were first fitted on complete cases with no dropout or other missing data (N). Model 2 was then fitted by using multiple imputation to impute missing data.

**Emotion recognition**

The estimated associations between medication use and emotion recognition scores are shown in Table 6 (higher scores represent a better performance). Under Model 1 which adjusts only for baseline covariates, neuroleptic users had worse performance on all emotion recognition tasks, and several of these associations were statistically significant. The direction of the association remained the same under Model 2 except for ‘happiness’, although became weaker on the whole. Similar results were found after accounting for missing data and three of the associations (neutral, sadness, surprise) remained borderline statistically significant. This provides some evidence that use of neuroleptics may impair emotion recognition.

There was no evidence of any association between use of SSRI/SNRIs and emotion recognition.

**Figure:**
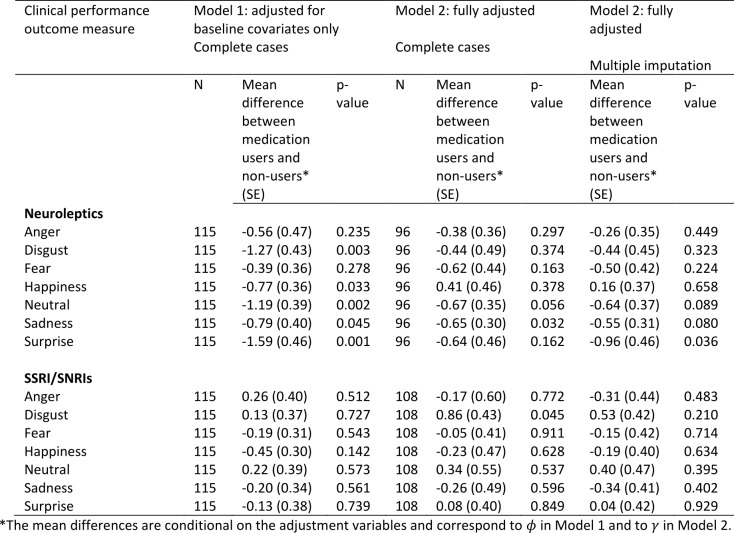
**Table 6:** Associations between medication use and emotion recognition scores using Model 1 (adjusted for baseline covariates only – sex, disease group, age, CAG, study site, education) and Model 2 (fully adjusted - for baseline covariates, prior medication use, use of other medications, prior performance measures). The models were first fitted on complete cases with no dropout or other missing data (N). Model 2 was then fitted by using multiple imputation to impute missing data.

**Neuropsychiatry**

The estimated associations between medication use and neuropsychiatry scores are shown in Table 7 (higher scores represent a worse performance). Adjusting only for baseline variables (Model 1), those taking neuroleptics had significantly higher (worse) apathy scores, but this disappeared after adjustment for prior medication use, use of other medications, and prior performance measures under Model 2. Neuroleptic use was associated with less depression as measured by the HADS total score. This improvement was in fact stronger and statistically significant under Model 2 compared with Model 1, and the effect remained strong and of borderline statistical significance after accounting for missing data.

After adjustment for baseline variables (Model 1) users of SSRI/SNRIs had worse scores for apathy, affect, irritability and total behaviour. However, after adjustment for confounding by prior medication use, use of other medications, and prior performance measures using Model 2, the direction of these associations was reversed, and the scores for apathy, affect and total behaviour were found to be statistically significantly lower in SSRI/SNRI users. The associations with apathy and total behaviour scores remained borderline statistically significant after accounting for missing data. The scores for affect and irritability were in the expected direction but not statistically significant.

Note that under Model 2 we adjusted for use of the other medication type in the models for neuroleptics and SSRI/SNRIs. Therefore the observed associations are independent of use of the other medication. Neuroleptic use was therefore associated with a borderline statistically significant lower (i.e. better) total HADS score independently of use of SSRI/SNRIs. Similarly, use of SSRI/SNRIs was associated with borderline statistically significant lower (i.e. better) scores for apathy and total behaviour independently of neuroleptics use.

**Figure:**
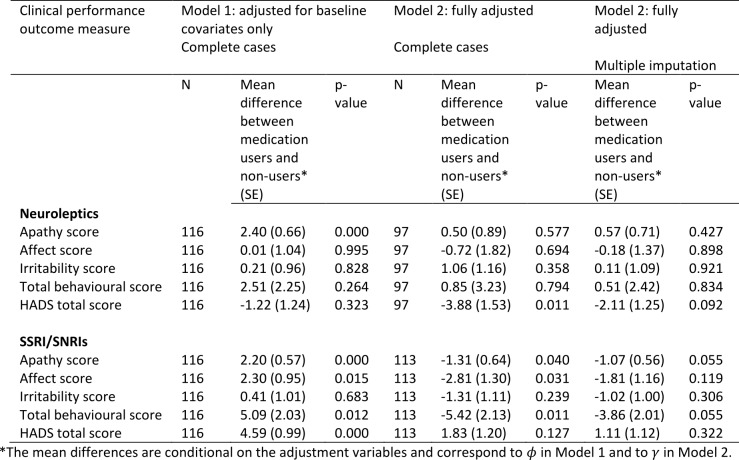
**Table 7:** Associations between medication use and neuropsychiatry scores using Model 1 (adjusted for baseline covariates only – sex, disease group, age, CAG, study site, education) and Model 2 (fully adjusted - for baseline covariates, prior medication use, use of other medications, prior performance measures). The models were first fitted on complete cases with no dropout or other missing data (N). Model 2 was then fitted by using multiple imputation to impute missing data.

## 4. Discussion

Using 36 months of data from early HD participants in TRACK-HD, we have studied associations between neuroleptics, SSRIs and SNRIs and performance on motor, cognitive and neuropsychiatric assessments. Our aim was to investigate whether therapies that are widely used in the symptomatic management of HD were likely to have concomitant effects not only on the symptom for which they were prescribed, but also for other endpoints. We adopted a statistical approach making use of the longitudinal nature of the data, using up to date methods and thinking on causality, accounting for prior medication use, use of other medications and prior clinical performance as potential confounding variables. In doing so, our aim was to come closer to a causal interpretation for estimated associations between use of medications and measures of clinical performance.

In the TRACK-HD cohort, the prevalence of SSRI/SNRI use was almost double that of neuroleptics at baseline, but use of neuroleptics increased at a greater rate during the 36 months of the study. Nevertheless, the type of medication was consistent from one study visit to the next in over 80% of cases, i.e. there were a higher number of new cases for neuroleptics, but once a participant was taking either neuroleptics or SSRI/SNRI it was probable that they would continue on the same medication until the next visit. Neuroleptic use at baseline was significantly greater in Paris participants, which may be due to regional or local prescribing practices, but there was no association between neuroleptics and disease stage. In contrast, use of SSRI/SNRIs was significantly more likely in those with stage 2 disease, with over half of participants in this group prescribed SSRI, SNRI or both, and use was consistent across sites.

Without adjustment for prior medication use and prior clinical performance, medication use was typically associated with worse concomitant outcomes on a number of measures, even after adjustment for demographic factors and disease severity; this at least in part reflects the fact that sicker patients tend to receive more medication. After adjustment for prior clinical performance, prior medication use and concomitant use of other medications many (but not all) of these “inverse” associations between medication use and clinical performance were eliminated. That is, participants who were taking neuroleptics at the time of assessment, tended to have better motor performance, poorer cognitive performance, and better affect; those taking SSRI/SNRIs had less apathy, less affect and better total behaviour scores. However, with the exception of the improvement in neuropsychiatric scores, we did not uncover many statistically significant associations.

Associations between neuroleptics and motor performance were particularly sensitive to adjustment for prior medication use, use of other medications, and prior performance measures. After adjustment for baseline variables only (sex, disease group, age, CAG, study site, education), those using neuroleptics had significantly worse motor performance. Ideally, neuroleptics are prescribed taking the risk-benefit into account where the benefit in reducing chorea is thought to outweigh the risks and other motor side effects. It is possible that neuroleptics were associated with a worsening of some motor symptoms even if there is an improvement in chorea. When the additional variables were taken into account we unveiled a statistically significant improvement in chorea in those taking neuroleptics and a tendency for improvement in grip force variability. However, the former result was non-significant after accounting for missing data, and there were no other measurable improvements compared to non-users. We note that we were not examining one agent in isolation and it is possible that some of the 18 different neuroleptics included here were less efficacious than others.

A limitation of this investigation, and likely an important reason why we did not find stronger associations, is the relatively small sample size, in particular relative to the number of variables adjusted for in order to account as far as possible for confounding using the data available. Furthermore, we must consider the possibility of unmeasured confounding. We cannot exclude the possibility that there are additional variables which confound the association between medication use and the clinical performance measures. For example, uncontrolled confounding may arise because participants within the neuroleptic or SSRI/SNRI groups differ in the severity of their symptoms or rate of disease progression, and thus their need for or response to medication, in a way not accounted for by the variables included in our analyses. Potential errors in measurements of clinical performance are a further limitation of this investigation and may have resulted in bias in our results, probably towards the null. Without additional information on any potential measurement errors it was not possible to make corrections for this. We were also lacking information on the reasons why a given medication was prescribed for a particular individual. Confounding may also occur because we cannot assume that neuroleptics were always prescribed for motor indications such as chorea. This may be broadly correct in a cohort of early HD participants such as those in TRACK-HD, but any HD group is also likely to contain at least some individuals who were prescribed neuroleptics for behavioural indications, especially irritability and aggression. In these cases the possible impact of a given medication on motor function is likely to be seen as a necessary consequence of controlling the antisocial behaviour. Although we collected information about indication during TRACK-HD, this was not standardised across sites until the 24-month visit and the data is therefore not considered suitable for this analysis. We had some limited information on duration of medication use, however it was not deemed appropriate to investigate the effects of duration of use given the quality of the data on this and the sample size.

In this investigation we studied the association between concomitant medication use and performance measures, with careful adjustment for confounding. The sample size was not sufficient to allow a more complex investigation into the impacts of longer term medication use on clinical performance. We also did not use information on individual medication doses or duration of use.

We had expected the association between medication and motor aspects of HD to be the easiest to demonstrate because it is arguably the most well documented to date. Current understanding of cognitive decline is largely focused on improving methods for measuring the earliest signs of onset and subsequent longitudinal change^2^^,^ 29. However, the direction of the associations we estimated did not contradict clinical observations that neuroleptics have an adverse impact on cognition, and the pattern of results for SSRI/SNRIs was broadly similar. As with the associations with motor performance, it is possible that we have not been able to uncover a statistically significant association due to the sample size or the large number of adjustment variables used under Model 2. However, as cognitive aspects of HD are less well documented, it is also possible that the association between neuroleptic or SSRI/SNRIs use and improvements in cognition in early HD is in reality not that strong.

After adjustment for baseline variables (Model 1) users of SSRI/SNRIs had worse scores for apathy, affect, irritability and total behaviour. However, after adjustment for confounders using Model 2, the direction of these associations was reversed, and the scores for apathy, affect and total behaviour were found to be significantly lower in SSRI/SNRI users. The associations with apathy and total behaviour scores remained borderline statistically significant after accounting for missing data. The scores for affect and irritability were in the expected direction but not statistically significant. This shows that with appropriate adjustment for confounding we can uncover anticipated associations between medication use and task performance.

Previous findings from TRACK-HD suggest that effects of medications on emotion recognition may need to become a relevant consideration in the pharmacological treatment of people with HD^9^. Similarly we showed here that neuroleptic use was associated with poorer recognition across the range of facial emotions, although only recognition of neutral and sad faces survived correction for confounding variables under Model 2, and we did not replicate the improvement seen previously in this dataset in SSRI/SNRI users. However, it is relatively well-established that emotion recognition, in particular recognition of negative emotions of disgust and anger^30^, are already impaired in HD so it is possible that additional deterioration in these emotions was difficult to measure in our sample after rigorous adjustment for confounding.

Previously in TRACK-HD, we have reported a consistent longitudinal increase in apathy in early HD relative to controls and have argued that this may be more easily observed than other neuropsychiatric symptoms such as depression and irritability as the latter are more treatable and therefore attenuated by pharmacological interventions^2^. Adjusting for baseline variables only, those taking neuroleptics had significantly worse apathy, which would be expected. However, this association was no longer present after adjustment for confounding variables. More surprisingly, we were not able to show from the current analysis that neuroleptics have any effect on irritability or aggression scores. This suggests that the number of participants in TRACK-HD with these indications may have been too few for the effect to be detectable. Interestingly, a significant decrease in the HADS score indicating an improvement in depression was observed under Model 2. Of further interest is that after adjustment for confounders using Model 2, scores for apathy and total behaviour were lower in SSRI/SNRI users, with borderline statistical significance, and scores for affect and irritability were in the expected direction but non-statistically significant after accounting for missing data.

Although, in general, associations between medication and performance of motor, cognitive and neuropsychiatric assessments were evident, the extent to which correction for confounding variables played a part in these associations varied between domains. In the case of motor performance, the direction of the association was reversed under Model 2 and became more clinically plausible, whereas the cognitive results were weaker after correction for potentially confounding variables. As discussed above this may reflect the strength of the original association as well as our sample size relative to the number of variables used in the statistical model, and the possibility of unmeasured confounding. The selection of variables for Model 2 should also be reviewed as there is some danger that the arguments become cyclic. For example, the relationship between TFC and our clinical measures is not necessarily causal. While it is conceivable, for example, that neuropsychiatric symptoms such as apathy and irritability, particularly if combined with cognitive decline, increase in response to loss of function, medication for neuropsychiatric symptoms may also directly impair functional capacity.

Despite using sophisticated statistical methods within a well-designed and controlled study, it has not been possible to establish completely clear findings regarding the impact of medication and performance in the various domains studied. Our interest was in the causal effects of medication, but medications are prescribed with the hope of improving physical and neurological manifestations of the disease so the outcomes of interest (motor scores, etc.) are in turn expected to affect medication. We used the longitudinal nature of the data to address this issue, though the potential for unmeasured confounding still remains. It would also be of interest in future investigations to consider duration of use. Ideally, the effects of medication use on performance in the domains studied in this paper would be investigated using double-blind, randomised, long-term studies assessing various treatment strategies. However, large observational data sets provide the possibility of performing such investigations, with due consideration given to the possible impact of unmeasured confounding, where randomized controlled trials have yet to be performed.

## 5. Supporting Information

**Figure:**
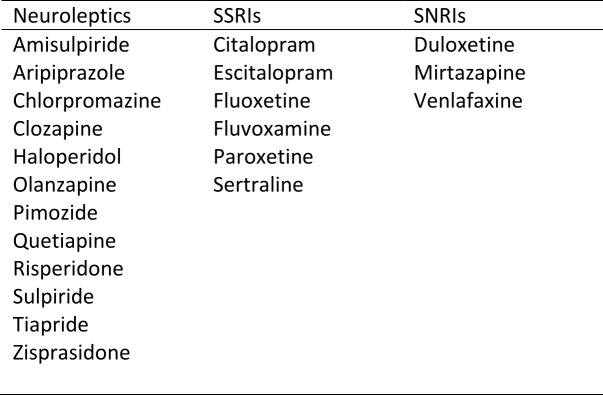
**Supplementary Table 1:** Drugs whose use was reported among early-HD participants in TRACK-HD according to their classification as neuroleptics, SSRI or SNRI.

**Figure:**
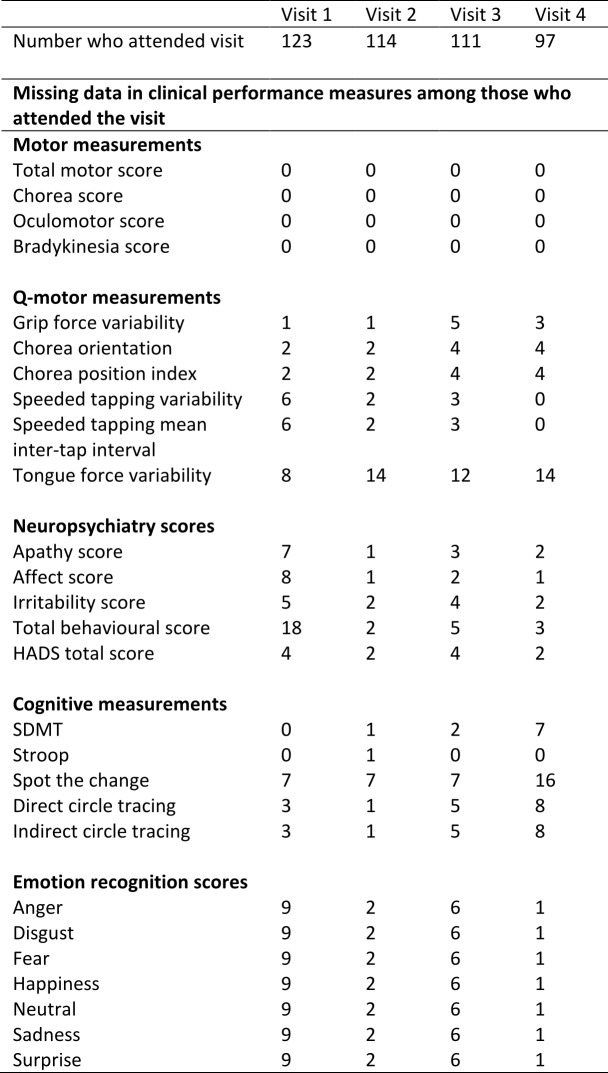
**Supplementary Table 2:** Summary of missing data among early-HD participants in Track-HD.
